# Mediator in the Embryo-endometrium Cross-talk: Granulocyte Colony-stimulating Factor in Infertility

**DOI:** 10.7759/cureus.5390

**Published:** 2019-08-15

**Authors:** Chris A Robert, Mohammed K Abbas, Abdul Rehman Z Zaidi, Suyeewin Thiha, Bilal Haider Malik

**Affiliations:** 1 Obstetrics & Gynecology, California Institute of Behavioral Neurosciences and Psychology, California, USA; 2 Internal Medicine, California Instititute of Behavioral Neurosciences and Psychology, California, USA; 3 Research, California Institute of Behavioral Neurosciences and Psychology, California, USA; 4 Internal Medicine, California Institute of Behavioural Neurosciences and Psychology, California, USA; 5 Medicine, California Institute of Behavioral Neurosciences and Psychology, California, USA

**Keywords:** infertility, recurrent implantation failure, thin endometrium, follicular fluid g-csf, g-csf, g-csf in infertility, rif, g-csf for thin endometrium, g-csf for recurrent implantation failure, g-csf for rif

## Abstract

Successful implantation requires a receptive endometrium and a good quality egg. The challenges a physician encounters with regard to this in assisted reproductive technology are obtaining good quality embryo, achieving optimal endometrial thickness (EMT), and subsequently implantation, which is denotive of a receptive endometrium. Granulocyte colony-stimulating factor (G-CSF) has been observed to be a biomarker of oocyte quality and has been shown to enhance EMT and implantation because of its immunological effects. A systematic search for all relevant articles on G-CSF in follicular fluid and its therapeutic benefit in thin endometrium and recurrent implantation failure was performed, and peer-reviewed, full-text articles related to humans were included in the study. As a tool to determine the potentiality of oocyte, G-CSF shows promise with its predictability increasing in combination with morphological embryo scoring or interleukin 15. For the thin endometrium, G-CSF is especially useful in patients who are refractory to other treatment modalities. In recurrent implantation failure (RIF), G-CSF showed potential in a subset of patients with immunological deficiency lacking killer cell immunoglobulin-like receptor genes. This review highlights the various forms of usage of G-CSF and the effectiveness of G-CSF in infertility. G-CSF equips embryologists with a tool to determine the potentiality of oocyte and physicians with therapy for thin endometrium and RIF, especially since the available treatment options are ineffective.

## Introduction and background

“Medicine is the restoration of discordant elements; sickness is the discord of the elements infused into the living body.”: Leonardo Da Vinci.

A 35-year-old woman presented with primary infertility with associated male-factor infertility of oligozoospermia for which intracytoplasmic sperm injection (ICSI) was recommended. During the first cycle of ICSI, although good follicular growth was observed, endometrial thickness (EMT) was 4 mm despite the use of estradiol and vasodilators. At the patient’s insistence, embryo transfer (ET) was performed, but it did not yield a positive result. For the second cycle, she opted for frozen embryo transfer (FET) to improve her chances of having a thicker endometrium. This time, even with proper estrogen priming of the endometrium, the thickness remained below 7 mm. The patient pressed for another option to improve her endometrium as she could not afford another ICSI cycle. Is granulocyte colony-stimulating factor (G-CSF) the answer?

Globally, 8% to 12% of reproductive-age couples suffer from infertility [1]. While the global average stands at 9%, it reaches 30% in some regions. Despite the advances in in vitro fertilization (IVF) and the technology to select a euploid embryo, 30% of the cases still do not result in a live birth [2].

Successful implantation depends on the embryo quality, receptive endometrium, and excellent ET technique [3]. Endometrial receptivity plays a vital role in the embryo-endometrium cross talk. EMT is essential in endometrial receptivity [4]. A meta-analysis demonstrated that reduced EMT leads to low pregnancy rates [5]. Mouhayar and Sharara reported that among the various available modalities, only G-CSF and vaginal sildenafil had a demonstrable impact on the endometrium [6]. G-CSF has gained considerable importance in recent years after an initial study by Gleicher et al. demonstrated successful treatment of thin endometrium, followed by several other researchers [7-8]. However, others have failed to show the same benefit in improving ET [9-11].

While thin EMT seems to be a determinant of endometrial receptivity, successful implantation is the test. The immunological etiology of many causes of recurrent implantation failure (RIF) explains why G-CSF could be useful in patients with RIF. While many researchers have studied the effect of G-CSF on thin endometrium, its benefit in patients with recurrent pregnancy loss and RIF is insufficiently proven [12]. Wurfel et al. demonstrated a positive effect in improving implantation rates in a subset of patients with immunological deficiency, those lacking killer immunoglobulin-like receptor (KIR) genes [12]. Other researchers have also shown improved implantation rates and clinical pregnancy rate [13-15]. However, questions of its efficacy arise because of the increased miscarriage rates found in some studies [12].

Moreover, G-CSF is also regarded as a biomarker of oocyte quality. Immunology plays a crucial role in implantation failure; hence, increased G-CSF in the follicular fluid is associated with an increased implantation rate [16-18]. Given its added advantages of being noninvasive and not affected by blood contamination, it has gained considerable research interest.

Despite all the advances in IVF, most clinicians are still unable to resolve problems on refractory endometrium and RIF. Various existing modalities proposed over the years have shown inconsistent results. G-CSF has shown promising results, but considering it as an absolute value in treating such problems remains uncertain. This article reviews all the published studies on the effectiveness of G-CSF for treating thin endometrium and RIF and as a diagnostic tool of oocyte quality. We examined whether G-CSF is associated with any substantial improvement in clinical pregnancy in patients with thin endometrium and RIF and if increased G-CSF values are associated with embryos with improved quality.

## Review

Methods

Search Method and Strategy

A computerized, systematic search was performed on April 17, 2019, using combinations of the following search keywords: granulocyte colony-stimulating factor, female infertility, thin endometrium, recurrent implantation failure, granulocyte colony-stimulating factor and female infertility, granulocyte colony-stimulating factor and implantation failure, granulocyte colony-stimulating factor and follicular fluid, granulocyte colony-stimulating factor, and thin endometrium. The MeSH keywords used were female infertility, granulocyte colony-stimulating factor, and granulocyte colony-stimulating factor with female infertility (Tables 1, 2).

**Table 1:** Keywords and combination of keywords used for the search

Regular Keyword	Database	Articles
Female infertility	PubMed	34206
Recurrent implantation failure	PubMed	4295
Thin endometrium	PubMed	255
Granulocyte colony-stimulating factor+ female infertility	PubMed	40
Granulocyte colony-stimulating factor + implantation failure	PubMed	33
Granulocyte colony-stimulating factor + follicular fluid	PubMed	26
Granulocyte colony-stimulating factor +thin endometrium	PubMed	11

**Table 2:** MeSH keywords and combination keywords used in the search

MeSH keyword	Database	Articles
Female infertility	PubMed	15143
Granulocyte colony-stimulating factor	PubMed	10000
Granulocyte colony-stimulating factor + Female infertility	PubMed	15

Study Selection

All study designs that investigated the significance of G-CSF in infertility-related pathologies, namely, thin endometrium and implantation failure, were included. To narrow down the search, we applied the inclusion and exclusion criteria. We included peer-reviewed, full-text articles related to humans, with participants involving females only regardless of age and location. Given that the review was concerned on a relatively new treatment modality, no year restriction was implemented. The abstracts of the chosen articles were studied, and only those with data deemed suitable for review were shortlisted. Furthermore, only literature articles in English language were analyzed to limit interpretation differences. The reference lists were also investigated to find other potentially eligible studies that can be included even if they were published more than five years ago.

Ethical Issues

In this study, patients were not directly involved because data were obtained from databases. Hence, ethical approval was not required.

Statistical Analysis

No statistical analysis was done as this is a traditional review.

Results

The literature search produced 80 relevant publications on PubMed database. The keywords G-CSF and infertility, G-CSF and implantation failure, G-CSF and thin endometrium, and G-CSF in follicular fluid yielded 34, 20, 13, and 13 articles, respectively.

A total of 1874 patients were included in the study, all of whom were females aged between 25 and 47 years. We found that G-CSF has been applied extensively in reproductive medicine. For instance, it is used as a tool for the selection of competent embryos and therapeutic applications in patients with thin endometrium and RIF.

Moreover, the literature review unveils that G-CSF is a sensitive biomarker of oocyte quality [19-20]. Unfortunately, the review does not provide a definitive answer, considering that the G-CSF’s feasibility in patients with polycystic ovary syndrome (PCOS) patients has conflicting results [21-22]. Additional studies are needed to ascertain if G-CSF is useful as a stand-alone tool or when combined with other methods, such as morphological embryo scoring [22] and even interleukin (IL)-15 quantification [18].

G-CSF in thin endometrium is an innovative approach, mainly because it can increase the EMT in approximately 48 hours [7]. Conversely, several other related modalities have unsatisfactory results [8]. The improvement in thickness in some studies may be possibly potentiated by sildenafil and aspirin [23]; others showed better results in combination with endometrial scratch [24]. However, these outcomes need to be explored further. Moreover, the varying doses, mode of administration, and day of administration used in various studies require a randomized control trial (RCT) to determine the optimal dosage to achieve the best results.

G-CSF in recurrent implantation failure (RIF) showed potential in a particular subset, those lacking KIR genes [19]. We have a long way to go in determining the cause and subsequent treatment options for RIF. A robust RCT might unlock the true potential of G-CSF in RIF.

Discussion

Implantation is a vital step during reproduction; the steps involved are apposition, attachment, and then the invasion of trophoblasts [25]. A cascade of cytokines, chemokines, and growth factors mediates the fetomaternal cross talk before fertilization, thereby facilitating implantation. For fertilization, a good-quality oocyte must come into contact with a low DNA damage sperm. The developing blastocyst must then interact with the endometrium, which is further aided by maternal immunological tolerance.

Uterine receptivity can be subdivided into three phases: prereceptive phase, receptive phase, and the nonreceptive phase. The receptive phase is in the midluteal phase, approximately 7-10 days after ovulation. If a competent embryo engages with the endometrium during this transient receptive phase, implantation ensues; if not, implantation fails because synchronization was not attained [26]. Trophoblastic invasion is regulated by locally produced immunological factors that maintain a balance of different immunological cell populations. Pregnancy is accompanied by the shift of the T helper cell type 1- T helper cell type (Th2) paradigm toward the Th2 cell type that predominates the basal plate of the placenta thwarting any attacks against embryo [27].

G-CSF is a glycoprotein synthesized by mononuclear cells (e.g., macrophages), fibroblasts, endometrial cells, and natural killer (NK) cells [12]. Locally produced G-CSF may also play a role in the modulation of cytotoxicity of NK cells and reduction of interferon-gamma (INF-g) and IL-8 [28]. G-CSF may additionally have a local angiogenic property, possibly due to the expansion of endothelial progenitor cells and proangiogenic gene expression in monocytes [16].

**Figure 1:**
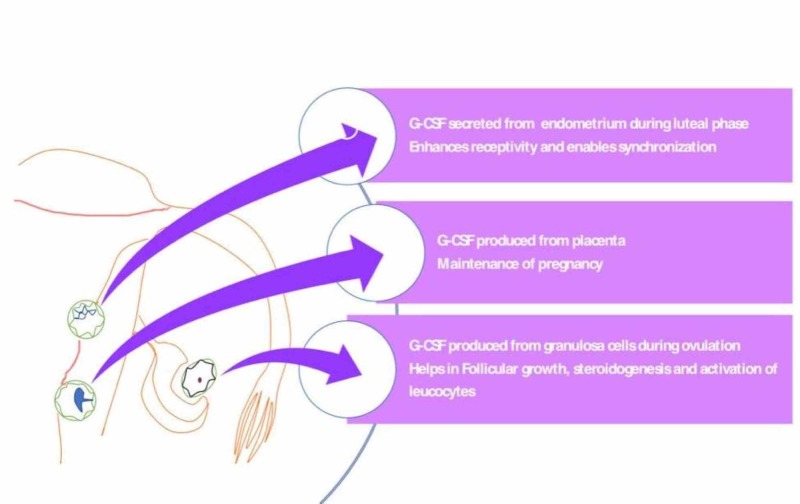
Sites of production of G-CSF in the reproductive tract G-CSF: granulocyte colony-stimulating factor

**Figure 2:**
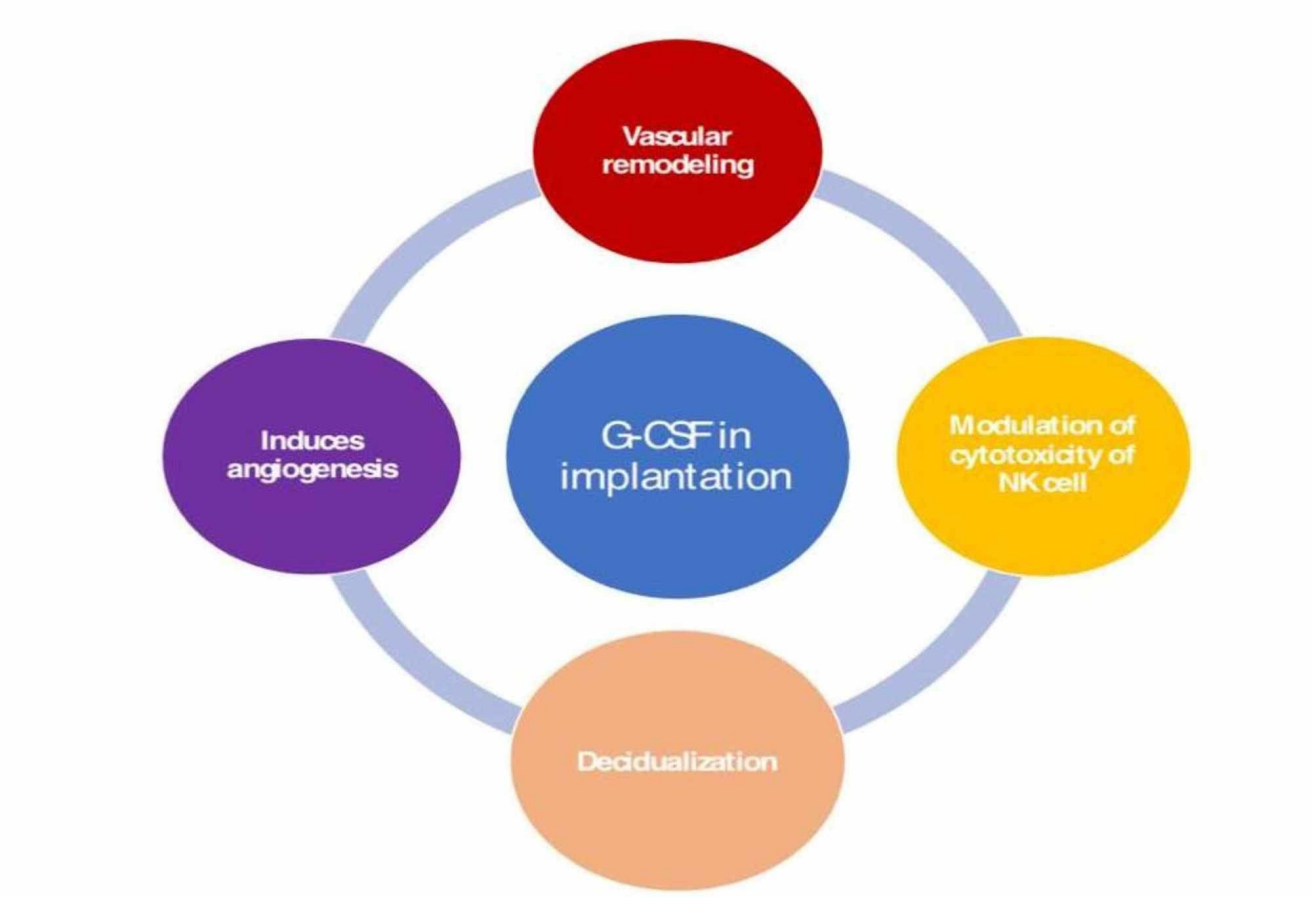
Role of G-CSF in implantation G-CSF: granulocyte colony-stimulating factor, NK: natural killer

In the reproductive tract (Figure 1), G-CSF is secreted in the following three ways: during ovulation by the granulosa cells, thereby promoting follicular growth, steroidogenesis, and activation of leucocytes necessary for ovulation; from the endometrium through the luteal phase, thereby inducing vascular remodeling and decidualization and finally, during gestation in the placenta, thereby favoring sustenance of pregnancy [29]. Moreover, G-CSF has a placental trophic effect and a function in embryonic development, although granulocyte-macrophage colony-stimulating factor (GM-CSF) is the main component involved. Thus, G-CSF exerts its influence at various levels of the implantation process, making it an attractive diagnostic and therapeutic tool [30] (Figure 2).

 

G-CSF Quantification in Follicular Fluid

One of the techniques to improve the probability of obtaining a live birth is culture prolongation through the blastocyst stage. However, with this method, we run the risk of ending up with no embryos to transfer on day 5 [31]. Another technique is to select the embryo according to morphological observation coupled with time-lapse imaging, but it still does not discriminate the potentiality of embryos [31]. G-CSF quantification in follicular fluid has been affirmed to be a useful biomarker of oocyte competence. It has distinct advantages. For instance, it is noninvasive, considering that it uses the readily available follicular fluid during oocyte retrieval, and it is not affected by blood contamination [31].

In the study of Ledee et al., 132 individual follicles exhibited significant differences in implantation rates between embryos with low (<20 pg/ml) and high (>24 pg/ml) G-CSF levels (9% vs. 44%, respectively) [32]. In their subsequent study involving 83 individuals, the birth rate in the group with a higher G-CSF level (38%) was significantly higher than that in the group with a lower G-CSF level (5%) [32]. A proof of concept study later undertaken afresh, revealed significantly high implantation rates, reaching 54% for embryos with optimal morphology and 37% for frozen-thawed embryos [31]. In combination with morphological embryo scoring on day 2, the predictability improved. Furthermore, Ledee et al. showed that a combination of G-CSF and IL-15 contributed to an improved predictability [33]. A different study found higher G-CSF levels in individuals with PCOS; however, the clinical pregnancy rates in patients with higher follicular-fluid G-CSF levels did not visibly improve [34]. Conversely, in a study by Niu et al., patients with PCOS and metabolic syndrome had significantly lower G-CSF levels compared with those with PCOS having no metabolic syndrome and those in the control group [21]. In addition, Gaafar et al. revealed that no significant difference was found in the G-CSF levels between patients with PCOS and those with other causes of infertility.

Most studies demonstrated that the effectiveness of G-CSF as a marker of successful implantation is possible, especially because it has a reasonable sensitivity rate. Compared with emerging techniques of genomic analysis of cumulus cells, G-CSF is simplistic and economical [19,35]. However, further RCTs are needed to reach a definitive conclusion on the suitability of G-CSF as a biomarker of oocyte quality.

G-CSF for Thin Endometrium

EMT is often regarded as a marker of endometrial receptivity. Though the usage of endometrial volume and Doppler sonography of the uterine and subendometrial blood flow as a predictive marker has gained traction in the recent years, EMT determination is still the most extensively used method in clinical practice [6-7]. In a study by Kasius et al., thin endometrium was observed in 2.4% of the total cases. The same study described a trend toward significantly lower pregnancy rates in patients with an EMT of less than 7 mm [5]. Despite having no consensus, an EMT of less than 7 mm has been generally accepted as suboptimal. Reduced EMT leads to lower pregnancy rates [5,20]. The systematic review and meta-analysis by Kasius et al. of 10,724 cases deduced that EMT is not an infallible predictor of pregnancy. However, the same study observed a significant drop in pregnancy rates in patients with an EMT of less than 7 mm [5]. Various approaches were tried to improve EMT, but they generally led to unsatisfactory results [25]. Mouhayar explored several treatment modalities, including extended estrogen, gonadotropin therapy, low-dose hCG, tamoxifen, pentoxifylline, tocopherol, L-arginine, low-dose aspirin, vaginal sildenafil, acupuncture and neuromuscular electric stimulation, intrauterine G-CSF, and stem cell therapy. Among the various available modalities, only G-CSF and vaginal sildenafil had an evident impact on the endometrium [6].

The endometrium has two layers, namely, the basal layer, which consists of large spiral arteries but remains intact during the menstrual cycle, and the functional layer, which is supplied by a thin capillary network that grows during the menstrual cycle and is entirely shed during menstruation. Following ovulation, the blood flow to the functional layer is reduced due to vasoconstriction of the spiral arteries. The resulting reduced oxygen tension aids implantation [36]. When the endometrium is thin, the embryonic implantation is closer to the basal layer with more blood flow and high oxygen tension, resulting in the generation of reactive oxygen species, which is detrimental to embryonic development and implantation [37]. G-CSF may act as a stimulant to the endometrial stem cells or mobilize the bone marrow stem cells to help expand the endometrium [38]. Furthermore, the local angiogenic property may be caused by an increase in endothelial progenitor cells and proangiogenic gene expression in monocytes [16,27].

The influence of G-CSF on a thin endometrium was studied originally by Gleicher et al. in a case series where an intrauterine infusion of G-CSF resulted in an increased EMT within 48-72 hours of infusion and a 100% pregnancy rate [7]. Their subsequent study, which included 21 patients, showed a statistically significant improvement in EMT, with a pregnancy rate of 19.1%. However, they were unsure if the increase was due to the synergistic effect of G-CSF and sildenafil, which was used in all patients [23]. While Kunicki et al. noted a significant improvement in EMT, the concomitant usage of aspirin and sildenafil may have had an additive effect [39]. Another RCT by Sarvi et al. showed that G-CSF had a positive influence on EMT but no improvement in the clinical pregnancy rate [40]. Xu et al. also conducted an RCT wherein G-CSF alone and G-CSF with endometrial scratch were compared with the control. The results revealed a significant increase in EMT in both G-CSF alone and G-CSF with endometrial scratch [41].

Please see Table 3 for a summary of all 12 studies.

**Table 3:** Studies on the effectiveness of G-CSF for thin endometrium EMT: endometrial thickness, ET: embryo transfer, hCG: human chorionic gonadotropin, OR: oocyte retrieval, FET: frozen embryo transfer, CPR: clinical pregnancy rate, IR: implantation rate

Author	Year	Sample size	Type of study	Age	Diagnostic criteria	Drug route	Admin time	Description of control	CPR	p-value	IR	p-value	EMT before treatment	EMT after treatment	P-value
Gleicher [7]	2011	4	Case series	33,34,41,45	Thin EMT <7 mm	300 micrograms into endometrial cavity	2-9 days before ET	-	100	NA	-	-	5.0 ± 1.2	8.6+-1.1	-
Gleicher [23]	2013	21	Prospective observational	40.5±6.6	Thin EMT <7mm	300 micrograms into endometrial cavity	6-12 hours before hCG trigger	Before treatment	19.1	NA	-	-	6.4 ± 2.1	9.3+-2.1	<0.001
Kunicki [39]	2014	37	Prospective cohort	34.68±4.13	Thin EMT <7 mm	300 micrograms into endometrial cavity	Before transfer	Before treatment	18.9	NA	-	-	6.74 ± 1.75	8.42 ± 1.73	<0.001
Eftekhar [42]	2014	68(34/34)	Non-randomised experimental study	30.81±4.60/28.57±5.16	Thin EMT <7 mm	300 micrograms into endometrial cavity	NA	No therapy	32.1/12	0.1	-	-	5.63 ± 0.78	7.91 ± 0.55	0.1
Shah [24]	2014	231	Observational cohort	33.48±3.79	Thin EMT <8 mm	300 micrograms into endometrial cavity	After 10 days of priming with oral estradiol and vaginal sildenafil	-	37/39.25	0.8272	-	-	7.98 ± 1.3	10.97 ± 1.23	<0.0001
Xu [41]	2015	82(30/52)	Prospective cohort	31.4±4.0/32.2±3.9	Thin EMT <7 mm	300 micrograms into endometrial cavity	Day follicle becomes dominant	Underwent FET despite thin EMT	41.8/25	0.038	31.5/13.9	<0.01	4.1 ± 0.9	8.1 ± 2.1	<0.001
Mishra [43]	2015	35	Prospective cohort	NA	Thin EMT <7 mm	300 micrograms into endometrial cavity	Day 14 of FET cycle	Before treatment	nil	NA	-	-	5.86 ± 0.58	6.58 ± 0.84	<0.01
Lee [44]	2016	50	Retrospective cohort	NA	Thin EMT <8 mm	300 micrograms into endometrial cavity	On day of OR or hCG trigger	-	22.00%	NA	-	-	7.2 ± 0.6	8.5 ± 1.5	<0.001
Sarvi [40]	2017	34	Randomised controlled trial	31.6 ± 3.8/31.2 ± 3.2	Thin EMT <6 mm	300 micrograms into endometrial cavity	On day of hCG adminstration	Normal saline	15.3/20	NS	103/5.4	0.001	4.1 ± 1.8	9.1 ± 1.5	0.001

However, other studies recorded no increase in EMT [10,42]. In an RCT conducted by Barad et al., patients with normal thickness endometrium did not show improvement in implantation or pregnancy rates, possibly attributed to the high mean age of the patients in the study [9]. Although Eftekar et al.’s study showed no increase in EMT, it presented improved implantation and clinical pregnancy rates [42]. This result can be explained by the fact that the increase of EMT is not the only effect G-CSF has on the endometrium; other physiological effects of G-CSF might have also occurred. Meanwhile, Li et al. reported that G-CSF did not have a positive impact on the EMT, although it had a significant effect on cycle cancelation rates [10]; the absence of a positive outcome may be explained by the lower doses used in the study.

G-CSF being a relatively new therapeutic method needs more RCTs to determine the ideal dosage, frequency, and day of administration. Additionally, RCTs are required to verify if G-CSF is indeed useful as a stand-alone tool or only when combined with other modalities, such as vasodilators, to improve the chances of implantation. The smaller number of currently available studies and the small sample size, along with the different study designs, made the findings difficult to interpret. Nonetheless, G-CSF shows promising results in its ability to significantly increase the EMT within 48-72 h and presents a trend toward higher implantation and pregnancy rates.

G-CSF in Recurrent Implantation Failure

Uterine NK (uNK) cell modulation is the cause of recurrent miscarriage, given that it is regarded as a biosensor of foreign antigens. Extravillous cytotrophoblast cells manifest nonclassical human leukocyte antigens, which interact with killer cell immunoglobulin-like receptors (KIRs) located in uNK cells. Consequently, uNK cell cytotoxicity is hindered, and angiogenic cytokine production increases [45].

Wurfel et al. conducted a study, but the inclusion was not only based on the history of repeated implantation failures; only patients lacking 3 KIR receptors were included. The incidence of this finding was extremely high, amounting to 78%. They showed a pregnancy rate of 42% for D2 transfer and 73.8% for D5. However, abortion rates were also high. They conducted a further pilot study wherein G-CSF was administered in patients with repeated IVF failures but did not lack the KIR gene, and the pregnancy rate was only 10% [12]. However, the study also showed an increased miscarriage rate, which seems to be countereffective. KIR gene profiling might have provided the answer, given that Wurfel et al. demonstrated the effectiveness of this treatment only in those deficient in KIR genes. Other studies have also shown a significant improvement in implantation rates [11,13,14] and increment in clinical pregnancy rates [11,13,15]. Research comparing the intrauterine and subcutaneous routes found the subcutaneous route to be more efficacious [46]. A summary of studies on the effectiveness of G-CSF in RIF can be seen in Table 4. In conclusion, G-CSF is a unique therapeutic method. However, it appears to be beneficial only in a subset of patients, especially those demonstrating immunological deficiency in the form of loss of KIR genes. Furthermore, the most effective mode of administration needs to be determined in further studies.

**Table 4:** Studies on the effectiveness of G-CSF for RIF SC: subcutaneous, IV: intravenous, ET: embryo transfer, hCG: human chorionic gonadotropin, FET: frozen embryo transfer, OR: oocyte retrieval, IR: implantation rate, CPR: clinical pregnancy rate, D2: day 2, D5: day 5 [12-15], [46-47]

Author	Year	Sample size (Test/Control)	Age (G-CSF vs Control)	Drug route	Time of administration	IR (Test/Contol/Placebo)	p-value	CPR (Test /Control/Placebo)	p-value	Chemical pregnancy (Test/Control)	p-value
Wurfel et al.	2010	59	NA	13 million units	Every three days	NA	NA	73.8%(D5)/42%(D2)/10	NA	NA	NA
Aleyasin et al.	2016	112	33.5±4.2/32.4±5.2	SC 300 micrograms	One hour before ET	18% vs. 7.2%	0.007	37.5% vs. 14.3%	0.005	44.6% vs. 19.6%	0.005
Davari-Tanha et al.	2016	80	35.5±4.32/35.3±3.98	IV infusion 300 micrograms	At the time of OR, in FET cycle the day of stating progesterone	12.3%/6.1/4.7	0.04	80/80/100	0.51	25/12.5/10	0.04
Eftekhar et al.	2016	90	32.55±4.61/31.75±5.16	IV infusion 300 micrograms	At the time of OR	16.67%/5.04%	0.0151	28.88% /13.3%	0.043	NA	NA
Obidniak et al.	2016	130	NA	SC 300 micrograms	Five days prior to ET or at the day of ET	31.2%/38.6%/19.8 - 19.8%	NA	37.5% /46.6% /26.6%	NA	NA	NA
Arefi et al.	2018	52	34.5±5.50/34.05±6.5	SC 300 micrograms	30 min before blastocyst transfer	NA	NA	56.2/40	0.09	NA	NA

G-CSF has many uses in diverse forms in relation to reproductive health. It may be utilized as a biomarker to determine oocyte quality and may enhance the EMT in thin endometrium and RIF. Consequently, pregnancy rates and live birth rates improve. From an immunological perspective, the role it plays in the abovementioned indications can be explained. However, particular areas of ambiguity exist; examples are the dosages and modes of administration and continuity of administration or even the idea if the effects of G-CSF were simply additive or possibly synergistic combined with other modules.

Limitations

When interpreting the conclusions, certain limitations should be considered. The included studies range from experimental studies to observational studies, including RCTs, nonrandomized experimental studies, and cohort studies. The heterogeneity of the studies made an accurate comparison of results impossible. Limitations also included the small sample size, along with different study designs, in many of the studies because G-CSF application is a relatively new concept. In addition, various researchers who tried out different dosages, modes of administration, and days of administration of G-CSF contributed to a weak comparison. The high cost of using G-CSF might have been responsible for the small sample size in the studies. For the review, we relied heavily on old literature for an explanation of the immunological processes, possibly indicating a gap in research that needs to be filled. Lastly, particular pertinent literature could not be accessed as a student because of an existing paywall.

## Conclusions

Ascertaining the G-CSF in the follicular fluid in each oocyte has its advantages such as noninvasiveness and sensitivity. The opportunity to choose only the most potential embryos averts the need for multiple IVF and the accompanying financial and psychological burdens. In combination with morphological embryo scoring at day 2, its predictability improves, thereby realizing the full potential of this tool. However, some disparity was observed in reaching conclusions regarding its usage in patients with PCOS. The expansion of the endometrium at approximately 48 hours in some patients is what makes G-CSF an excellent therapeutic tool. Insight into whether the same effects would be seen if G-CSF was used alone or its best usage would be achieved when it is combined with aspirin, sildenafil, or endometrial scratch. Considering that endometrial volume and Doppler sonography of uterine and subendometrial blood flow are better determinants of endometrial function or even endometrial receptivity array, more studies are needed to assess these endometrial markers with the use of G-CSF. G-CSF in RIF shows its effectiveness only in a subset of patients with immunological deficiency lacking KIR genes. This review highlights the various forms of the usage and effectiveness of G-CSF in infertility while emphasizing on the multiple gaps in the literature and further recommendations for research. 
